# Thermal frequency shift and tunable microwave absorption in BiFeO_3_ family

**DOI:** 10.1038/srep24837

**Published:** 2016-04-20

**Authors:** Yong Li, Xiaoyong Fang, Maosheng Cao

**Affiliations:** 1 School of Material Science and Engineering, Beijing Institute of Technology, Beijing 100081, China; 2 School of Science, Yanshan University, Qinhuangdao 066004, China

## Abstract

Tunable frequency is highly sought-after task of researcher, because of the potential for applications in selecting frequency, absorber, imaging and biomedical diagnosis. Here, we report the original observation of thermal frequency shift of dielectric relaxation in La/Nd doped BiFeO_3_ (BFO) in X-band from 300 to 673 K. It exhibits an unexpected result: the relaxation shifts to lower frequency with increasing temperature. The relaxation maximally shifts about a quarter of X-band. The nonlinear term of lattice vibration plays an important role in the frequency shift. The frequency shift leads to tuning microwave absorption, which almost covers the whole X-band by changing temperature. Meanwhile, the great increase of dielectric loss of La/Nd doped BFO due to thermal excited electron hopping enhances microwave absorption above ~460 and ~480 K, respectively. The microwave absorption of La/Nd doped BFO surpasses −20 dB at 673 K, and the minimum reflection loss of La doped BFO reaches −39 dB. These results open a new pathway to develop BFO-based materials in electromagnetic functional materials and devices for tunable frequency, stealth and thermal imaging at long wavelength.

Technological tuning frequency has long been sought in absorbers applied in a variety of fields^1^,^2^,^3^. Typical strategies for tuning frequency are activating arrays and designing special configuration in composites. For instance, metamaterials have acquired frequency tunability by micromachined reconfiguration^4^, or by incorporating active components^5^,^6^,^7^. Nano-materials have carried out frequency tenability by controlling dimensions of nanoparticles and stacking nanosheets with different intersectional angles^8^,^9^. However, for common bulk absorbers, although high-efficiency and broad frequency have been achieved by various technologies^10^,^11^,^12^,^13^,^14^,^15^, tuning frequency is still got through the only way, changing the thickness of the absorber layer^16^. The way is very discommodious for the absorbers to tune frequency in practical applications. Even the thickness to meeting the particular frequency can exceed the thickness limit of absorber layer. Tuning frequency of the absorbers by an easy and feasible way, therefore, is still highly challenging. It is quite necessary to search for a new absorber, which possesses sensitive frequency response to external stimulation, such as light, electricity, magnetism or temperature.

BiFeO_3_ (BFO) is a fascinating multifunctional material with unique physical properties^17^,^18^,^19^. Moreover, the dielectric of BFO and doped BFO exhibits featured frequency response in microwave field due to its distorted structure and defect^20^. The amount of doping also has effect on dielectric properties. These are beneficial to tuning microwave absorption of BFO. In this work, we focus on the dielectric and microwave absorption properties La/Nd doped BFO with doping amount of 0.2, which possess more excellent microwave absorption than that of other doping amounts. The frequency shift of relaxation and the tunability of microwave absorption at elevated temperature are investigated.

## Results and Discussion

**Figure 1:**
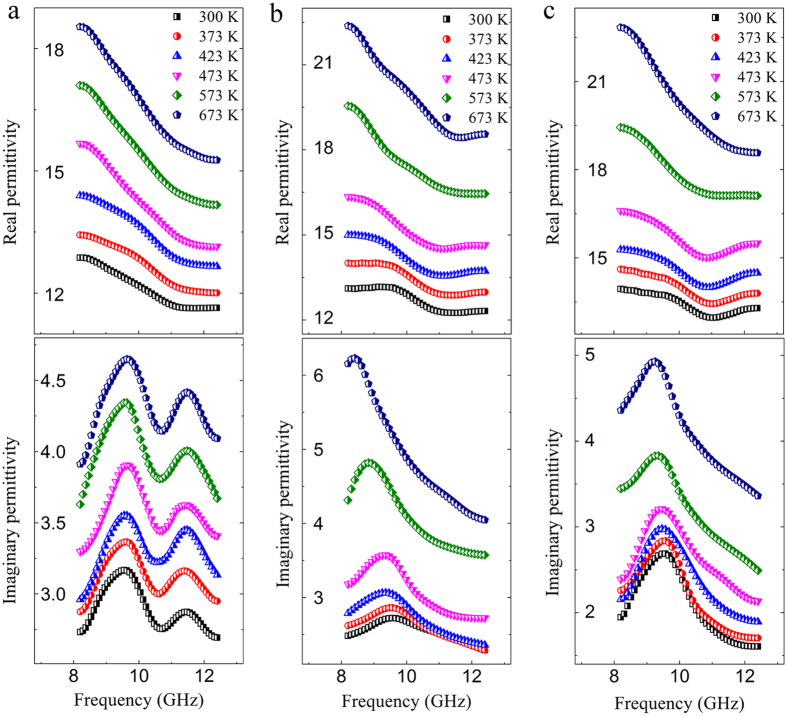
The complex permittivity of (**a**) BFO, (**b**) La doped BFO and (**c**) Nd doped BFO in X-band from 300 to 673 K.

The dielectric properties were measured in microwave frequency range (X-band, 8.2–12.4 GHz) from 300 to 673 K by the high-temperature waveguide test apparatus. The real and imaginary permittivity versus frequency at elevated temperature is shown in Fig. 1. The real permittivity of BFO and La/Nd doped BFO in this study decreases with increasing frequency, and increases with increasing temperature, demonstrating dispersion response over the full X-band and strong temperature dependence. The relaxation peaks are found in imaginary permittivity of all samples, which exhibit dielectric relaxation behavior. The imaginary permittivity increases with increasing temperature for all samples. BFO exhibits a slow growth over the investigated temperature, while La/Nd doped BFO have an abrupt rise at ~460 and ~480 K, respectively (Fig. S1).

**Figure 2:**
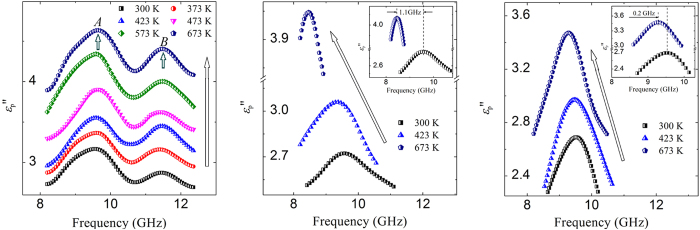
The polarization part (*ε*_p_”) versus frequency in (**a**) BFO, (**b**) La doped BFO and (**c**) Nd doped BFO. The insets show the distance of relaxation shift from 300 K to 673 K. The arrows represent the shift direction of relaxation peak with increasing temperature.

**Figure 3:**
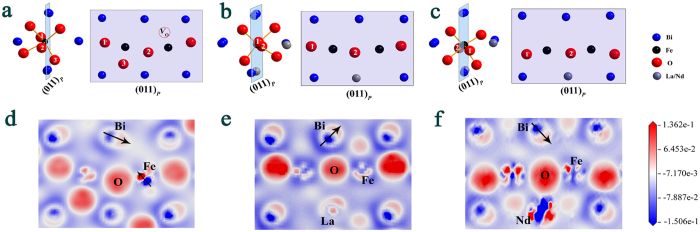
The structure of (**a**) BFO, (**b**) La doped BFO and (**c**) Nd doped BFO. The difference charge density of (**d**) BFO, (**e**) La doped BFO and (**f**) Nd doped BFO.

The imaginary permittivity consists of conduction part (*ε*_c_”) and polarization part (*ε*_p_”). According to the conductivity (Fig. S2), the frequency dependence of *ε*_c_” is acquired (Fig. S3). The *ε*_c_” of La/Nd doped BFO is bigger than that of BFO due to the abrupt rise of the conductivity above ~460 and ~480 K, respectively (Fig. S2). The electron hopping plays an important part in the great increase of conductivity^21^,^22^. The *ε*_p_” of all samples versus frequency from 300 to 673 K are shown in Fig. 2. After subtracting *ε*_c_” from imaginary permittivity, the dielectric relaxation is obviously observed in the *ε*_p_” of all samples. It is noted that two relaxations (relaxation *A* and relaxation *B*) appear in BFO, which are located respectively at ~9.6 GHz and ~11.8 GHz, hardly shift with increasing temperature. For La/Nd doped BFO, however, only one relaxation appears in the frequency range, and shifts to low frequency with increasing temperature (the whole data in Fig. S4). The relaxation of La doped BFO shifts 1.1 GHz and the relaxation of Nd doped BFO shifts 0.2 GHz (inset in Fig. 3b,c).

Figure 3a–c. show the structure of BFO and La/Nd doped BFO in a pseudocubic crystal plane (001)_*p*_. The X-ray diffraction (XRD) and transmission electron microscopy (TEM) show that BFO is single phase perovskite with rhombohedral structure, while La doped BFO and Nd doped BFO possess pseudotetragonal structure^23^ and the PbZrO_3_-like structure^24^, respectively (Fig. S5 and S6). The structural evolution is attributed to the substitution of La/Nd for Bi, which weakens the hybridization of Bi 6*s* and O 2*p* orbits, leading to the decrease of the distortion of BFO (Fig. S7)^17^. Figure 3d–f show the difference charge density corresponding to (011)_*p*_. For BFO, the charge asymmetric distributions appear in Bi sites and Fe sites adjacently to oxygen vacancy (*V*_O_). For La/Nd doped BFO, the charge asymmetric distributions only appear in Bi ion sites. The charge asymmetric distributions Bi sites and Fe sites in BFO are respectively caused by the lone pair of Bi ion and *V*_O_, which form intrinsic dipole and defect dipole. The X-ray photoelectron spectroscopy (XPS) shows the *V*_O_ concentration of La/Nd doped BFO is much less than that of BFO (Fig. S8). Thus La/Nd doped BFO only possess intrinsic dipole in Bi sites. Therefore, it is believed that the relaxation *A* and relaxation *B* in BFO are induced by the intrinsic dipole polarization and defect dipole polarization, respectively. The relaxation in La/Nd doped BFO is induced by intrinsic dipole polarization only.

With increasing temperature, the two relaxations of BFO hardly shift, while the relaxation of La/Nd doped BFO shifts to low frequency. Actually, the relaxation behavior is related to lattice vibration energy at elevated temperature. In brief, lattice vibration energy is related to temperature, including zero-point energy, linear term and nonlinear term. Based on the calculated results (Tab. S1), for BFO, the lattice vibration energy is just dominated by linear term, thus the relaxation time is independent on temperature (Fig. S9) and the relaxations hardly shift. For La/Nd doped BFO, the nonlinear term is bigger than that of BFO. The nonlinear term depends on temperature. The relaxation time increases with increasing temperature, thus the relaxation shifts to low frequency.

La/Nd doping gives rise to increase of imaginary permittivity and shift of the relaxation at elevated temperature which provide potential application for La/Nd doped BFO in microwave absorption. The microwave absorption of all samples is evaluated based on the complex permittivity. The reflection loss (*RL*) at a given frequency and layer thickness can be calculated as:


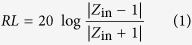


**Figure 4:**
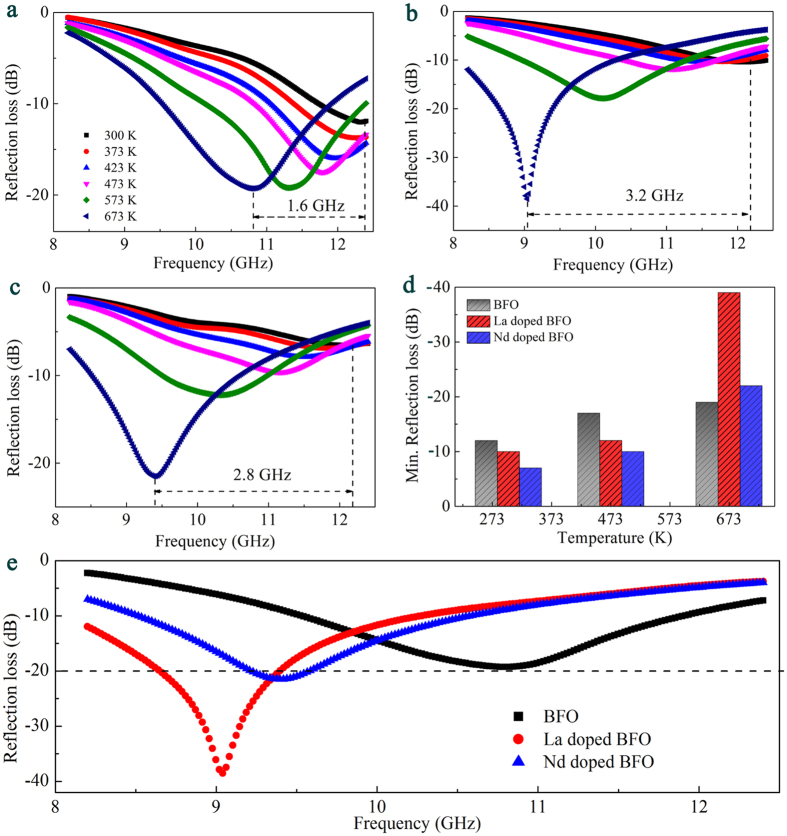
The reflection loss of (**a**) BFO, (**b**) La doped BFO and (**c**) Nd doped BFO. (**d**) The minimum reflection loss of BFO and La/Nd doped BFO at different temperature. (**e**) The reflection loss of BFO and La/Nd doped BFO versus frequency at 673 K.

Here the normalized input impedance *Z*_in_ of the electromagnetic attenuation layer is given by:


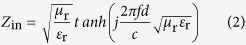


where *c* is the light velocity, *f* the electromagnetic wave frequency, *d* the thickness of the absorber, *ε*_r_ the complex permittivity, and *μ*_r_ the complex permeability. Figure 4a–c show the *RL* of all samples with thickness (*d*) of 1.8 mm at 300–673 K. With increasing temperature, the absorption peak shifts to low frequency. The absorption peak of BFO shifts ~1.6 GHz from 300 K to 673 K. The absorption peak of La doped BFO and Nd doped BFO shifts ~3.2 GHz and ~2.8 GHz, respectively. This indicates that La/Nd doped BFO can tune absorption frequency in much wider frequency range by thermal driving. The tunable feature of microwave absorption is attributed to the shift of the relaxation by varying the temperature. With increasing temperature, the *RL* of all samples increases. Compared with BFO, La/Nd doped BFO possesses stronger microwave absorption at high temperature. The minimum (Min.) *RL* of La doped BFO reaches −39 dB at 673 K, which is 2 times higher than that of BFO (Fig. 4d). The Min. *RL* of Nd doped BFO surpasses −20 dB at 673 K (Fig. 4e). The increase of microwave absorption for La/Nd doped BFO at high temperature is attributed to the increase of imaginary permittivity. It is found that the *ε*_c_” increases respectively above ~460 and ~480 K due to the increase of hopping conduction, which plays an important role in the increase of imaginary permittivity (Fig. S10). Therefore, the ability of varying temperature to tune absorption frequency and enhanced microwave absorption indicate that La/Nd doped BFO may be high-efficiency and smart absorber.

In summary, the dielectric and microwave absorption properties of BFO and La/Nd doped BFO at 300–673 K are investigated. La/Nd doped BFO demonstrate that the relaxation shifts to low frequencies by thermal driving at elevated temperature, which is closely related to lattice vibration energy. The imaginary permittivity of La/Nd doped BFO greatly increases above ~460 and ~480 K due to the increase of electron hopping. The shift of relaxation and increase of imaginary permittivity enhance the ability of tuning absorption frequency and intensity. The absorption peak of La doped BFO and Nd doped BFO respectively shifts ~3.2 GHz and ~2.8 GHz, which increases by 100% and 75% compared with that of BFO. The Min. *RL* of La/Nd doped BFO surpasses −20 dB at 673 K, where the Min. *RL* of La doped BFO reaches −39 dB, 2 times higher than that of BFO. This work highlights the application of BFO-based materials as smart absorber in microwave fields.

## Methods

### Experimental details

Sol–gel method was employed to prepare BiFeO_3_, Bi_0.8_La_0.2_FeO_3_ and Bi_0.8_Nd_0.2_FeO_3_ nanoparticles. Bismuth nitrate (Bi(NO_3_)_3_·5H_2_O) and iron nitrate (Fe(NO_3_)_3_·9H_2_O) as raw materials in stoichiometric proportions (1:1 molar ratio) were dissolved in 2-methoxyethanol (C_3_H_8_O_2_). By adding 2-methoxyethanol and nitric acid, the solution was adjusted to a pH value of ~4. Then citric acid in 1:1 molar ratio with respect to the metal nitrates was added to the solution, followed by polyethylene glycol as a dispersant. The mixture was stirred for 30 min at 50 °C to obtain the sol, and then it was kept at 80 °C for 48 h to form the dried gel. The dried gel was calcined at 300 °C. The calcined powders were sintered at 500 °C for 2 h, and then cooled rapidly to room temperature.

The structure of the samples was collected using X-ray power diffraction (XRD, Ni-filtered Cu K_*α*_ radiation, 40 kV). Rietveld refinement of the XRD patterns was performed by using the FullProf Program. The diffraction profile was modeled by the Thompson–Cox–Hastings pseudo-Voigt function. The morphologies and microstructure were determined by transmission electron microscopy (TEM, JEOL-2100, Japan). Raman spectra were obtained using Raman spectromater (HORIBA, Jobin Yvon HR800). XPS spectra were measured on a PHI Quantera system with a C60 ion gun. The DC conductivity measurements of the samples (with painted silver electrodes) were carried out using an Keithley 2401A-6517B multi-meter. The complex permittivity was measured on an Anritsu 37269D vector network analyzer by the waveguide method in X-band from 300 to 673 K, where the samples were pressed into pellets with dimensions of 23.10 mm × 10.30 mm × 2.20 mm and then annealed at 500 °C for 30 min.

### First-principles calculations

The calculations on difference charge density were performed using the CASTEP program code based on the first-principles plane-wave pseudo-potential method. The generalized gradient approximation (GGA) was adopted along with the exchange-correlation function realized by Perdow-Burke-Emzerhof (PBE). The plane wave cutoff energy of 500 eV, the convergence criteria for energy of 2 × 10^−5^ eV, SCF tolerance of 2 × 10^−6^ eV and 2 × 2 × 2 *K*-point Monkhorst-Pack grid were applied to guarantee a well-converged structure under study. A 2*a* × 2*b* × c supercell of BiFeO_3_ was adopted for all the calculations.

## Additional Information

**How to cite this article**: Li, Y. *et al*. Thermal frequency shift and tunable microwave absorption in BiFeO_3_ family. *Sci. Rep.* **6**, 24837; doi: 10.1038/srep24837 (2016).

## Supplementary Material

Supplementary Information

## References

[b1] Watts C. M., Liu X. L. & Padilla W. J. Metamaterial Electromagnetic Wave Absorbers. Adv. Mater. 24, OP98–OP120 (2012). 22627995 10.1002/adma.201200674

[b2] Calame J. P. & Abe D. K. Applications of advanced materials technologies to vacuum electronic devices. P. IEEE. 87, 840–864 (1999).

[b3] Ren F. J. . Current progress on the modification of carbon nanotubes and their application in electromagnetic wave absorption. RSC Adv. 4, 14419–14431 (2014).

[b4] Fu Y. H. . A micromachined reconfigurable metamaterial via reconfiguration of asymmetric split-ring resonators. Adv. Funct. Mater. 21, 3589–3594 (2011).

[b5] Chen H. T. . Active terahertz metamaterial devices. Nature 444, 597–600 (2006). 17136089 10.1038/nature05343

[b6] Chen H. T. . Experimental demonstration of frequency-agile terahertz metamaterials. Nat. Photonics 2, 295–298 (2008).

[b7] Shrekenhamer D., Chen W. C. & Padilla W. J. Liquid crystal tunable metamaterial absorber. Phys. Rev. Lett 110, 177403 (2013). 23679774 10.1103/PhysRevLett.110.177403

[b8] Wang M. . Truncated spherical voids for nearly omnidirectional optical absorption. Opt. Express 19, 20642 (2011). 21997074 10.1364/OE.19.020642

[b9] Sun H. . Cross-stacking aligned carbon-nanotube films to tune microwave absorption frequencies and increase absorption intensities. Adv. Mater. 26, 8120–8125 (2014). 25338951 10.1002/adma.201403735

[b10] Wang G. Z. . Microwave absorption properties of carbon nanocoils coated with highly controlled magnetic materials by atomic layer deposition. ACS Nano 6, 11009–11017 (2012). 23171130 10.1021/nn304630h

[b11] Tong G. X. . Generalized green synthesis and formation mechanism of sponge-like ferrite micro-polyhedra with tunable structure and composition. Nanoscale 6, 778–787 (2014). 24257742 10.1039/c3nr03745b

[b12] Liang C. Y. . SiC-Fe3O4 dielectric-magnetic hybrid nanowires: controllable fabrication, characterization and electromagnetic wave absorption. J. Mater. Chem. A 2, 16397–16420 (2014).

[b13] Kuang J. L. & Cao W. B. Stacking faults induced high dielectric permittivity of SiC wires. Appl. Phys. Lett. 103, 112906 (2013).

[b14] Song N. N. . Integrating giant microwave absorption with magnetic refrigeration in one multifunctional intermetallic compound of LaFe_11.6_Si_1.4_C_0.2_H_1.7_. Sci. Rep. 3, 2291 (2013). 23887357 10.1038/srep02291 PMC3724178

[b15] Zhao T. K. . Electromagnetic wave absorbing properties of amorphous carbon nanotubes. Sci. Rep. 4, 5619 (2014). 25007783 10.1038/srep05619 PMC4090627

[b16] Zhang X. F., Huang H. & Dong X. L. Core/shell metal/heterogeneous oxide nanocapsules: the empirical formation law and tunable electromagnetic losses. J. Phys. Chem. C 117, 8563–8569 (2013).

[b17] Catalan G. & Scott J. F. Physics and Applications of Bismuth Ferrite. Adv. Mater. 21,2463–2485 (2009).

[b18] Seidel J. . Conduction at domain walls in oxide multiferroics. Nat. Mater. 8, 229–234 (2009). 19169247 10.1038/nmat2373

[b19] Yang C. H. . Electric modulation of conduction in multiferroic Ca-doped BiFeO_3_ films. Nat. Mater. 8, 485–493 (2009). 19396162 10.1038/nmat2432

[b20] Li Y., Cao W. Q., Yuan J., Wang D. W. & Cao M. S. Nd doping of bismuth ferrite to tune electromagnetic properties and increase microwave absorption by magnetic–dielectric synergy. J. Mater. Chem. C 3, 9276–9282 (2015).

[b21] Wen B. . Temperature dependent microwave attenuation behavior for carbon-nanotube/silica composites. Carbon 65, 124–139 (2013).

[b22] Wen B. . Reduced graphene oxides: light-weight and high-efficiency electromagnetic interference shielding at elevated temperatures. Adv. Mater. 26, 3484–3489 (2014). 24648151 10.1002/adma.201400108

[b23] Yuan G. L., Or S. W. & Chan H. L. W. Structural transformation and ferroelectric-paraelectric phase transition in Bi_1−x_La_x_FeO_3_ (x = 0–0.25) multiferroic ceramics. J. Phys. D: Appl. Phys. 40, 1196–1200 (2007).

[b24] Karimi S., Reaney I. M., Levin I. C. & Sterianou I. Nd-doped BiFeO3 ceramics with antipolar order. Appl. Phys. Lett. 94, 112903 (2009).

