# Supplementary material for: Thermal frequency shift and tunable microwave absorption in BiFeO3 family
Source: Sci Rep. 2016 Apr 20;6:24837. doi: 10.1038/srep24837 (PMC4837409; doi:10.1038/srep24837)
Supplement: Supplementary Information [file srep24837-s1.pdf]

Supplementary information

Thermal frequency shift and tunable microwave absorption in $BiFeO_3$ family

Yong Li[1], Xiaoyong Fang[2] & Maosheng Cao[1]

[1]School of Material Science and Engineering, Beijing Institute of Technology, Beijing 100081, China

[2] School of Science, Yanshan University, Qinhuangdao 066004, China

**Correspondence should be addressed to M. S. Cao (email: caomaosheng@bit.edu.cn).*

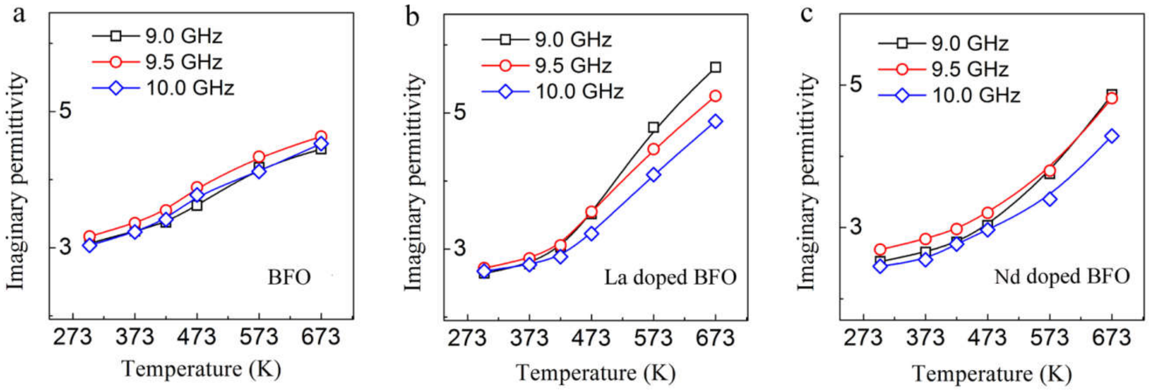

Fig. S1 The temperature dependence of the imaginary permittivity.

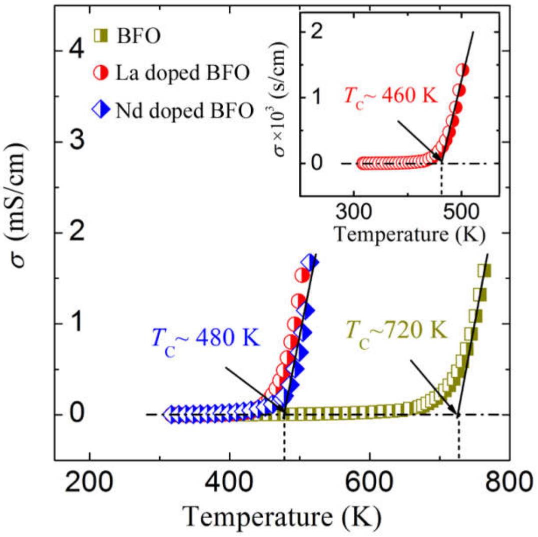

Fig. S2 The conductivities (σ) versus temperature in BFO, La/Nd doped BFO. Above the critical temperature (T_C), the electrons can hop by thermal driving in field. Therefore, the conduction of La/Nd doped BFO is dominated by electron hopping conduction above T_C, and the conductivity greatly increase with increasing temperature.

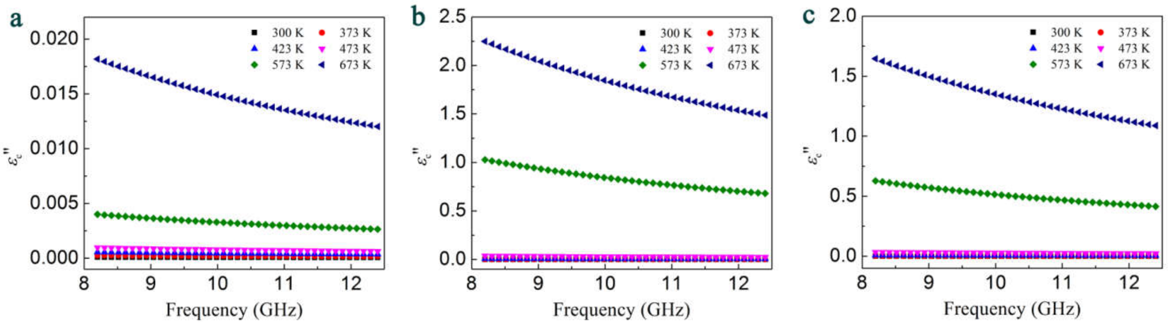

Fig. S3 The conduction part (ε_c'') of (a) BFO, (b) La doped BFO and (c) Nd doped BFO.

For solid medium, the imaginary permittivity is represents as Debye equation:

$$\varepsilon'' = \frac{(\varepsilon_s - \varepsilon_\infty)\omega\tau}{1+\omega^2\tau^2} + \frac{\sigma}{\varepsilon_0\omega} \quad (1)$$

Where ε_s is the static permittivity, ε_∞ is high-frequency permittivity, ω is angular frequency, τ is polarization relaxation time and σ is conductivity of medium. Here, the conduction part [$\varepsilon_c'' = \sigma/(\varepsilon_0\omega)$] can be obtained based on the experimental conductivity.

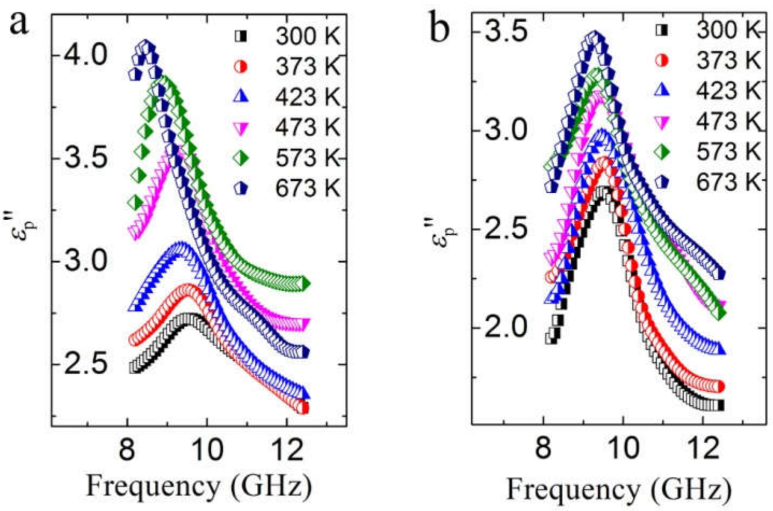

Fig. S4 The polarization part (ε_p'') of (a) La doped BFO and (b) Nd doped BFO. The polarization part (ε_p'') was gained by subtracting the ε_c'' from the imaginary permittivity.

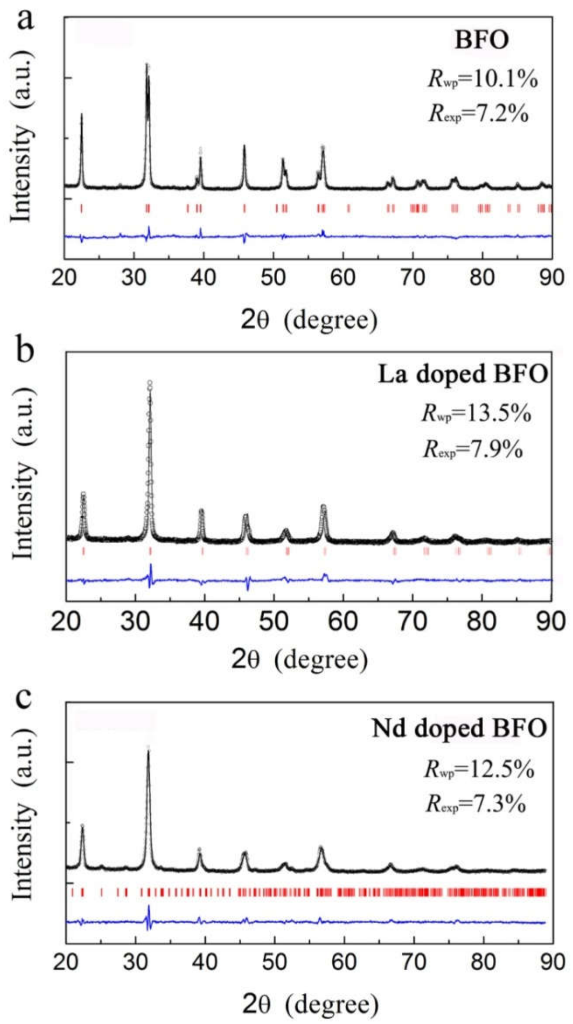

Fig. S5 XRD patterns of BFO and La/Nd doped BFO at room temperature. Experimental (circle), calculated (continuous line), and difference (bottom line) XRD profiles by Rietveld refinement. According to XRD scans, BFO possesses a rhombohedral crystal structure. Since La^{3+}, Nd^{3+} is doped into BFO respectively, La doped BFO and Nd doped BFO exhibit pseudotetragonal structure and the $PbZrO_3$-like structure, respectively.

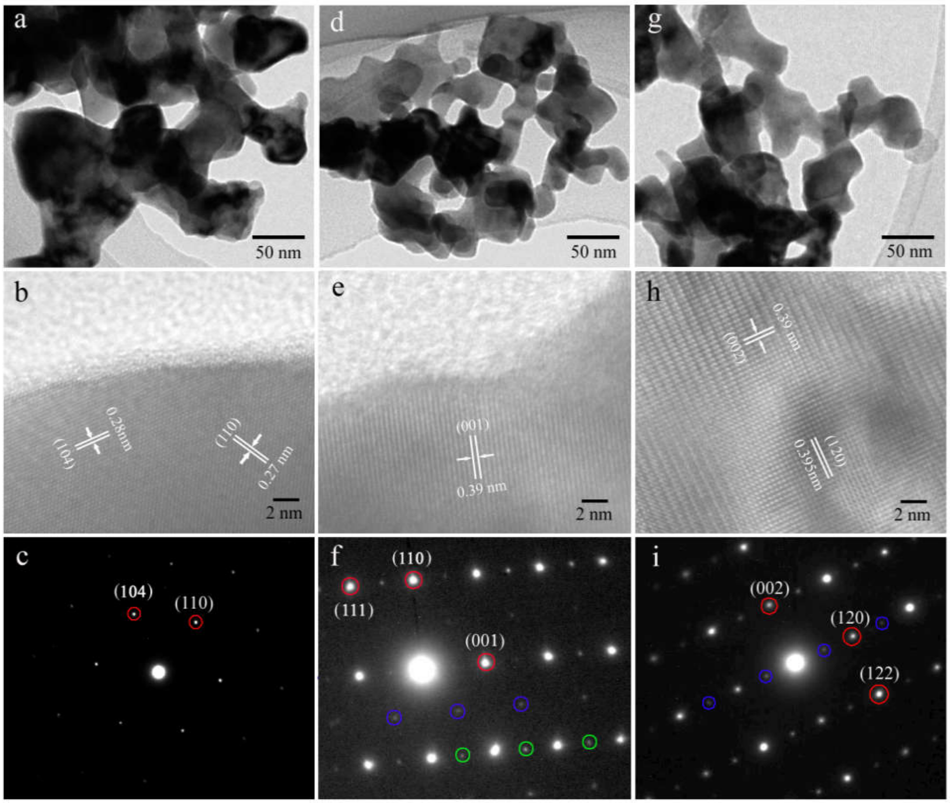

Fig. S6 The TEM images of BFO and La/Nd doped BFO nanoparticles. Morphology and grain size of BFO (a), La doped BFO (d) and Nd doped BFO (g). The high resolution TEM (HRTEM) images and interplanar spacing (white line) of BFO (b), La doped BFO (e) and Nd doped BFO (h). The selected area electron diffraction (SADE) patterns of BFO (c), La doped BFO (f) and Nd doped BFO (i), showing the corresponding lattice plane (red circles), two dominant 2-fold superstructures (blue circles and green circles) for BLFO and a 2-fold superstructures (blue circles) for BNFO. It is observed that the particles of three samples are at nanoscale sizes. For La doped BFO, the SAED shows the (001), (110) and (111) crystal planes of the pseudotetragonal structure. The superlattice reflection of $^1/_2$(001) and $^1/_2$(111) type is clearly observed. Nd doped BFO exhibits the $^1/_2$(120) type superlattice reflection. The results indicate the structural modulation in La/Nd doped BFO which is probably due to cations and oxygen vacancy ordering.

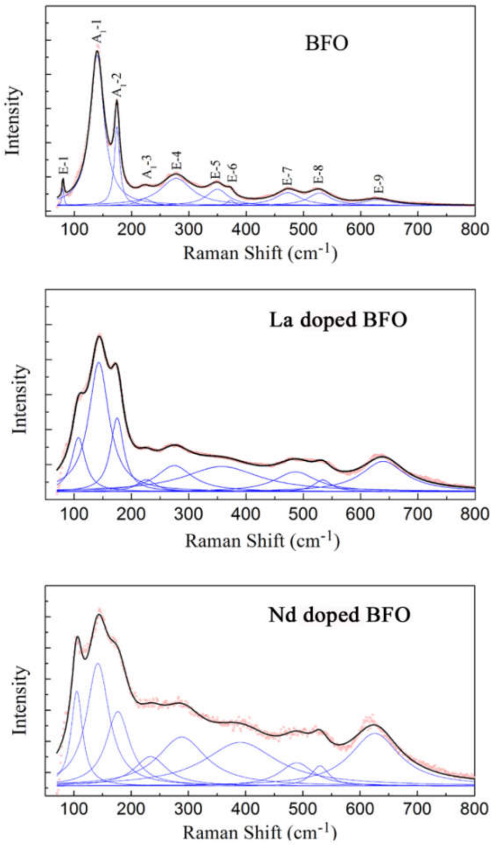

Fig. S7 Raman spectra of BFO and La/Nd doped BFO at room temperature. The circle, black line and blue line represent Experimental Raman spectra, calculated Raman spectra and peak-fitting simulations. Raman spectroscopy results show that *E*-1 mode nearly disappears, and the intensity of A_1-1, A_1-2 and A_1-3 weaken, where the modes are related to the Bi–O covalent bonds. This indicates that the hybridization of Bi 6*s* and O 2*s*/2*p* orbital weakens, leading to the structural evolution of BFO duo to La/Nd doping.

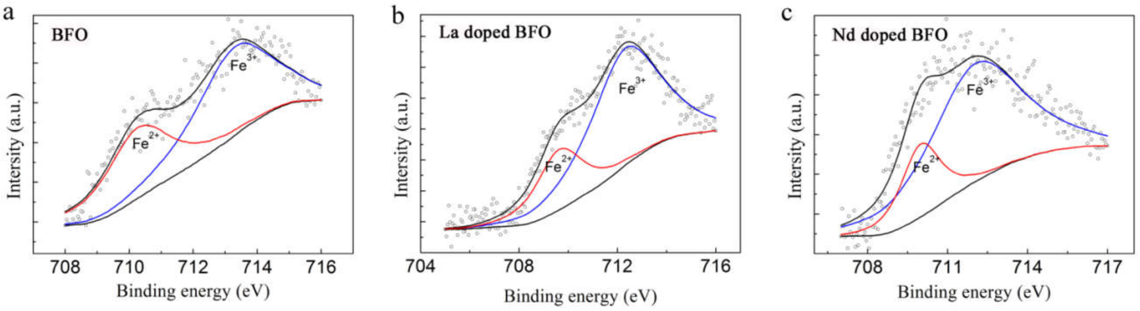

Fig. S8 High-resolution XPS of Fe $2p_{3/2}$ core levels for BFO and La/Nd doped BFO. Circle represents the experimental data. Black line represents the fitting data; Blue and red lines represent the peak-fitting simulations of Fe^{3+} and Fe^{2+}, respectively. The percentage of Fe^{2+} decrease significantly after La/Nd doping. It is known that Fe^{2+} and oxygen vacancies appear simultaneously for charge compensation in BFO materials. This result implies that the concentration of oxygen vacancies decrease after doping.

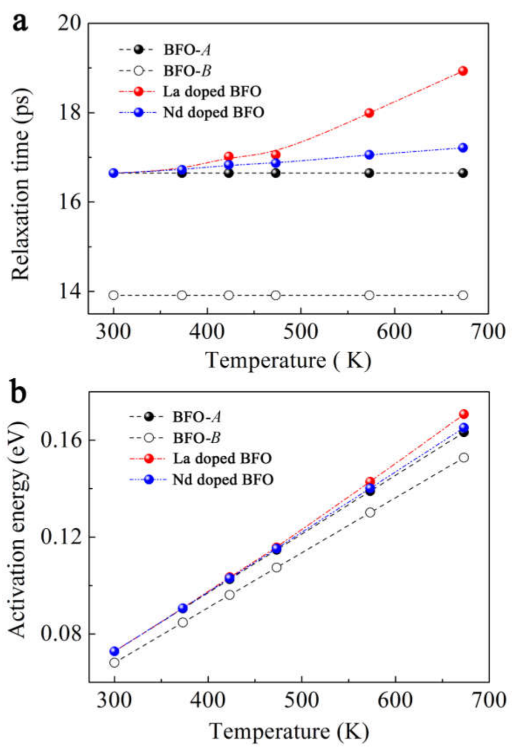

Fig. S9 (a) The relaxation time and (b) activation energy of the relaxation in BFO and La/Nd doped BFO versus temperature. The relaxation time of the relaxation in BFO hardly changes with increasing temperature. For La/Nd doped BFO, the relaxation time of the relaxation increases with increasing temperature. In general, the relaxationtime is as following:

$$\tau(T)=\tau_0\exp\left(\frac{W}{k_\mathrm{B}T}\right) \quad (2)$$

where τ_0 is a pre-exponential factor, W is the activation energy, determined by lattice vibration energy (U). Lattice vibration energy is related to $k_\mathrm{B}T$. And U can be expressed as:

$$U(k_BT)=U(0)+\left.\frac{\partial U}{\partial(k_BT)}\right|_{k_BT=0}(k_BT)+\left.\frac{\partial^2U}{\partial(k_BT)^2}\right|_{k_BT=0}(k_BT)^2+\cdots=U_0(0)+U_1(T)+U_2(T)+\cdots \quad (3)$$

where U_0, U_1, U_2 are zero-point energy, linear term and nonlinear term, respectively. Generally, the relaxationtimeis not only related to temperature, but also related to lattice vibration energy. Hence, the relaxationtimecan be expressed as:

$$\tau(T)=\tau_0\exp\left(\frac{U_0+U_1+U_2+\cdots}{k_BT}\right) \quad (4)$$

U_1 and U_2 are relative to the relaxation mode, lateral elasticity coefficient (C) and the linear expansion coefficient (β_T) in crystal, as well as temperature.

For BFO, the U_1 is much bigger than U_0 and U_2 in orientation polarization and ion relaxation polarization

(Tab. S1), thus the U_0 and U_2 are neglected. Considering U_1 increase linearly with increasing temperature, the relaxationtime is independent on temperature，thus the relaxation hardly shift. For La/Nd doped BFO, Bi-O bond becomes weaken, they possess bigger linear expansion coefficient. Meanwhile, considering La/Nd doped BFO show bigger lateral elasticity coefficient, thus the U_2 play major role on the relaxationtime.It is noted that the square relationship between U_2 and temperature, the relaxation time increases with increasing temperature, leading to the relaxation shift to low frequency.

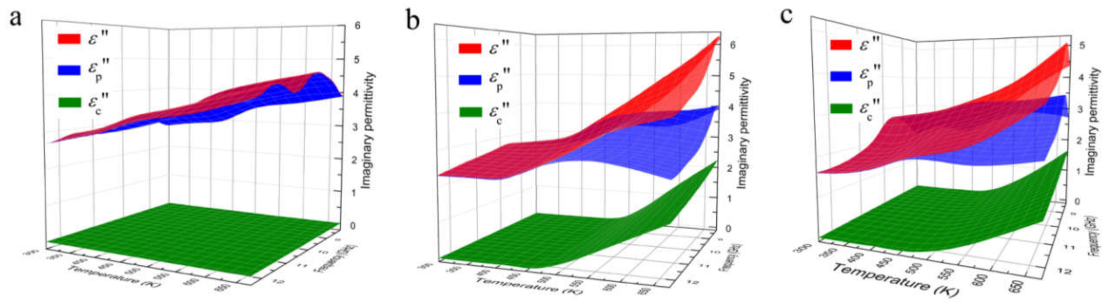

Fig. S10 The three-dimensional plots of the ε'', ε_p'' and ε_c'' of (a) BFO, (b) La doped BFO and (c) Nd doped BFO versus temperature and frequency.

Tab. S1. Lattice vibration energy in BFO and La/Nd doped BFO from 300K to 673K

	Relaxation	U_0(10^{-4} eV/ Å^3)	U_1(10^{-4} eV/ Å^3)					U_2(10^{-4} eV/ Å^3)				
			300K	373K	473K	573K	673K	300K	373K	473K	573K	673K
BFO	*A*	1.656	0.695	0.882	1.122	1.336	1.603	0.003	0.005	0.008	0.011	0.013
	B		20.84	25.92	32.86	39.81	46.76	0.267	0.534	0.802	1.336	1.656
La doped BFO	--	1.667	64.64	80.50	102.0	124.4	145.5	1.626	3.252	4.879	8.131	10.25
Nd doped BFO	--	1.697	62.98	57.66	99.38	120.2	141.1	1.636	3.272	4.908	7.361	9.897
